# Adverse Childhood Experiences Associated with Greater Internalization of Weight Stigma in Women with Excess Weight

**DOI:** 10.3390/obesities1010005

**Published:** 2021-06-03

**Authors:** Natalie G. Keirns, Cindy E. Tsotsoros, Samantha Addante, Harley M. Layman, Jaimie Arona Krems, Rebecca L. Pearl, A. Janet Tomiyama, Misty A.W. Hawkins

**Affiliations:** 1 Department of Psychology, Oklahoma State University, 116 Psychology Building, Stillwater, OK 74078, USA; 2 Department of Clinical and Health Psychology, University of Florida, 1225 Center Drive, Gainesville, FL 32610, USA; 3 Center for Weight and Eating Disorders, Department of Psychiatry, Perelman School of Medicine, University of Pennsylvania, 3535 Market Street, Suite 3108, Philadelphia, PA 19104, USA; 4 Department of Psychology, University of California, A623 Franz Hall, 501 Portola Plaza, Los Angeles, CA 90095, USA

**Keywords:** weight stigma, internalized weight stigma, adverse childhood experiences, ACEs, childhood abuse, obesity

## Abstract

Adverse childhood experiences (ACEs) may be an early life factor associated with adult weight stigma via biological (e.g., stress response), cognitive (e.g., self-criticism/deprecation), and/or emotional (e.g., shame) mechanisms. This pilot study investigated relationships between ACEs and internalized and experienced weight stigma in adult women with overweight/obesity and explored differential relationships between weight stigma and ACE subtypes (i.e., abuse, neglect, household dysfunction). Adult women (68% white, *M*_age_ = 33 ± 10 years, *M*_BMI_ = 33.7 ± 7.2 kg/m^2^) completed measures of ACEs (ACE Questionnaire), internalized weight stigma (IWS; Weight Bias Internalization Scale—Modified; WBIS—M), and lifetime experiences of weight stigma (yes/no). Data were analyzed with linear and logistic regression (*n* = 46), adjusting for age, race, and body mass index (BMI). Linear regressions revealed a positive association between ACE and WBIS—M scores (*β* = 0.40, *p* = 0.006), which was driven by Abuse-type ACEs (*β* = 0.48, *p* = 0.009). Relationships between WBIS—M scores and Neglect- and Household-Dysfunction-type ACEs did not reach significance (*β* = 0.20, *p* = 0.173; *β* = −0.16, *p* = 0.273). Though descriptive statistics revealed greater rates of experienced weight stigma endorsement by those with high-3+ ACEs (81%) vs. medium-1–2 ACEs (67%) or low/no-0 ACEs (60%), ACE scores were not significantly associated with experienced weight stigma in logistic regression (Wald = 1.36, *p* = 0.244, OR = 1.324, 95%, CI = 0.825–2.125). ACEs may be an early life factor that increase the risk for internalizing weight stigma in adulthood. Larger studies should confirm this relationship and follow-up on descriptive findings suggesting a potential association between ACEs and experienced weight stigma.

## 1. Introduction

Weight stigma, or the “social devaluation and denigration of people who are perceived to carry excess weight [1]” is endemic to Western societies [2]. While weight stigma is often directly experienced, it also may be internalized (i.e., applied to oneself); internalized weight stigma (IWS) is characterized by self-devaluation based on weight [3]. Experiencing and internalizing weight stigma is associated with consequences to both physical health (i.e., poorer overall health, mortality risk) and psychological well-being (i.e., depression, body dissatisfaction) [1,4–7]. Given these highly detrimental effects, it is imperative to understand risk factors for both experiencing and internalizing weight stigma.

One such risk factor may be adverse childhood experiences (ACEs)—chronic and pervasive events that occur in childhood, including experiences of abuse, deprivation or neglect, and household dysfunction [8]. The chronic stress of ACEs has been consistently related to biological changes which increase appetite and visceral fat accumulation, and ACEs are a well-established risk factor for both the development of adult obesity and for increasing severity of adult obesity levels [9,10]. Thus, these factors may put individuals with ACEs at risk for experiencing more weight stigma [11]. Furthermore, ACEs—and particularly experiences of childhood abuse—are linked to attention biases for threatening information [12], which could impact how individuals identify and perceive stigmatizing experiences when they are encountered. In other words, ACEs history may increase the likelihood that an individual will recognize instances of discriminatory or unfair weight-based treatment and identify them as stigmatizing. Still further, like weight stigma, ACEs are linked to the use of unhelpful coping strategies specific to eating (e.g., binge eating) and negative cognitive and emotional patterns, such as tendencies toward self-criticism and shame [10,13–17]. As self-criticism and shame center on negative self-evaluation, they are closely related to devaluing oneself based on weight or IWS [18–21]. Therefore, a proposed negative cycle may develop in which individuals experiencing ACEs are more likely to have greater obesity and consequent risk for experienced weight stigma. They may also be more likely to identify these stigmatizing experiences and to engage in maladaptive, obesogenic coping behaviors when under stress. Maladaptive coping responses and/or other’s criticisms may intersect with a tendency toward self-critical thoughts and feelings of shame, which may manifest as IWS, further exacerbate stress levels, and lead to continued maladaptive coping strategies and potential weight gain. Importantly, in this cycle, the development of IWS can be independent of both objective obesity and experienced weight stigma, as IWS is driven by self-perceived weight and self-application of weight-based stereotypes, and, therefore, can occur in the absence of excess weight and/or experienced weight stigma.

The hypotheses inherent to this cyclical model have not yet been tested, but preliminary evidence supports the proposed associations between ACEs and weight stigma. A small number of studies have identified a link between ACEs—and/or childhood maltreatment more generally—and weight stigma in adulthood [18,19,22]. Udo and Grilo [22] found that women with a history of childhood maltreatment (i.e., abuse or neglect) were more likely to report experiencing weight-based discrimination in the past year, and Braun et al. [18,19] observed positive correlations between ACEs and both experienced and internalized weight stigma in bariatric surgery patients. However, no study to date has investigated whether ACEs are robustly associated with IWS in broader populations or after considering related covariates (e.g., BMI). Understanding whether or not objective obesity level impacts the relationship between ACEs and weight stigma is important because—as mentioned above—many negative effects of weight stigma are linked to one’s self-perceived weight status and can occur in the absence of objectively measured overweight/obese BMI [23].

It is additionally unknown whether there may be differential associations of ACE types (i.e., abuse, neglect, household dysfunction) with weight stigma, as there is some evidence of abuse and neglect-type ACEs being more detrimental than household dysfunction for adult outcomes [24]. For instance, there is a more robust literature linking childhood abuse to feelings of shame in adulthood compared to the other ACE subtypes [25–29]. This evidence base may implicate pathways that contribute to the proposed relationship between ACEs and IWS by way of abuse leading to self-deprecation or negative self/body image.

The current pilot study takes initial steps in filling these gaps regarding the ACEs–weight stigma relationship by providing a preliminary investigation of the following two aims: (1) to explore whether a history of ACEs is associated with internalization of weight stigma, and (2) to explore whether a history of ACEs is associated with lifetime experiences of weight stigma. Secondary objectives include exploring potential differential associations of ACE types (e.g., abuse, neglect, household dysfunction) on internalized or experienced weight stigma.

## 2. Materials and Methods

### 2.1. Participants

Participants were adult women with overweight/obesity enrolled in the Neurotrophic Indicators of Cognition, Executive Skills, Plasticity, and Adverse Childhood Experiences Study (NICE SPACES), a pilot project examining weight, neurocognitive health, and stress reactivity in women with and without a history of ACEs (ClinicalTrials.gov, accessed on 2 June 2021, Identifier: NCT04076722). The primary aims of NICE SPACES were to assess the roles of brain health and ACEs in stress reactivity among women with excess adiposity. Participants were recruited from the university campus and surrounding community via email and social media. Participants with high (3+) and low/no (0) ACEs were preferentially recruited to increase ACEs variability, but individuals of all ACE levels were enrolled in the study, including “medium” ACE levels (1–2 ACEs). Eligibility criteria relevant to the current project include the following: (1) BMI ≥ 25 kg/m^2^, (2) English-speaking, (3) no use of weight-loss medications in the past 3 months, (4) no history of bariatric surgery, (5) not currently pregnant or breastfeeding, (6) not currently enrolled in a weight-loss program, and (7) no significant medical or psychiatric comorbidities.

### 2.2. Measures

#### 2.2.1. Adverse Childhood Experiences (ACEs)

An expanded version of the Adverse Childhood Experiences Questionnaire was used to assess the occurrence of ACEs prior to participants’ eighteenth birthday [8,30]. This ACE Questionnaire assesses for the 10 traumatic childhood events identified by Felitti and colleagues [8] (i.e., physical, emotional, or sexual abuse (Abuse-type ACEs); emotional or physical neglect (Neglect-type ACEs); parental separation/divorce; parental domestic violence; parental incarceration; drug or alcohol use in the household; or mental illness or attempted suicide in the household (Household Dysfunction-type ACEs)) with 17 yes/no questions, resulting in possible total scores ranging from 0–17. This expanded, 17-item measure allows for a more nuanced assessment of the standard 10 ACEs. For example, the traditional questionnaire assesses for emotional abuse with one item asking, “Did a parent or other adult in the household often or very often push grab, slap, or throw something at you? Or ever hit you so hard that you had marks or were injured?” whereas the 17-item measure breaks this into two questions: “Did a parent or other adult in the household often push, grab, slap, or throw something at you?” and “Did a parent or other adult in the household ever hit you so hard that you had marks or were injured?” Additionally, unique effects of the three subtypes of ACEs (i.e., abuse, neglect, household dysfunction) [31] were explored. A list of the expanded ACEs Questionnaire items and their groupings into subscales can be found in Supplementary Materials: Table S1.

#### 2.2.2. Internalized Weight Stigma (IWS)

The Weight Bias Internalization Scale—Modified (WBIS—M) was used to measure IWS [32,33]. The WBIS—M consists of 11 items that measure the extent to which individuals apply negative stereotypes to and devalue themselves because of their weight (e.g., “*My weight is a major way that I judge my value as a person*”). Response options range from 1 (*Strongly Disagree*) to 7 (*Strongly Agree*), and scores are represented by a mean of all responses. Higher average scores are indicative of greater internalization of weight stigma. In the current study, the WBIS—M displayed good reliability (α = 0.85), similar to the findings of the original validation sample (α = 0.94) [32].

#### 2.2.3. Experienced Weight Stigma

To assess whether participants had ever experienced weight stigma, questions were adapted from Puhl, Luedicke, and Heuer (2011) [34]. In a series of three questions, participants were asked to indicate (yes/no) if they had *ever* been teased, treated unfairly, or discriminated against because of their weight. Responses of “yes” were coded as 1 and responses of “no” were coded as 0. Paralleling previous literature, a dichotomous measure of whether participants had ever experienced weight stigma was calculated as 1 (“yes” on at least one of the three items) or 0 (“no” to all three items) [35,36].

#### 2.2.4. Covariates

Measured BMI and demographic variables (i.e., age, race) were included as covariates. BMI was calculated as kg/m^2^ from height and weight measured using a research-grade TANITA body composition analyzer. Demographic factors were collected via an online self-report questionnaire. Race was coded as follows: 1 = American Indian/Alaska Native, 2 = Asian, 3 = Native Hawaiian or Other Pacific Islander, 4 = Non-Hispanic Black or African American, 5 = Non-Hispanic White, 6 = White—Hispanic, 7 = Multiracial. Due to insufficient representation in each group, racial categories were dummy coded as 0 = historically oppressed/marginalized racial group and 1 = non-Hispanic white for analyses.

### 2.3. Procedure

All participants completed an online pre-screener survey in order to qualify for the study. Following enrollment, participants completed an at-home online survey and a 3 h in-lab assessment and were compensated 60 USD. ACEs, demographic variables, IWS, and lifetime-experienced weight stigma were assessed via online surveys. BMI was measured in-lab by a trained research assistant. All procedures were approved by the university’s IRB and adhered to APA ethical guidelines. Participants provided informed consent prior to study initiation.

### 2.4. Data Analysis Plan

All data were verified and checked for normality prior to analyses. Study aims were analyzed using linear or logistic regression. ACE subtype scores were entered simultaneously into regression models and multicollinearity checks (tolerance > 0.1, VIF < 10) were conducted to ensure the appropriateness of each model. Covariates in all analyses were age, race, and BMI; any non-significant covariates were retained in final models due to theoretical justification for their inclusion. All analyses were conducted in SPSS Version 25.

## 3. Results

### 3.1. Participant Characteristics

A total of 53 women (68% white, *M*_age_ = 33 ± 10 years) enrolled in the study. All women had a measured BMI in the overweight or obese range (≥25.0 kg/m^2^, *M*_BMI_ = 33.7 ± 7.2 kg/m^2^). Among these participants, 51 responded to the ACE questionnaire: 11 (22%) endorsed 0 ACEs, 17 (33%) endorsed 1 or 2 ACEs, and 23 (45%) endorsed 3 or more ACEs. In the full sample, the most commonly endorsed ACE type was household dysfunction (60.4%), followed by abuse (52.8%) and neglect (34.0%). An additional five participants were excluded from analyses due to missing data on other key variables. Therefore, 46 participants were included in the analyzed sample. Table 1 includes participant characteristics for the full sample and by ACE group status.

### 3.2. Aim 1: Internalized Weight Stigma

#### 3.2.1. Total ACE Score

Linear regression was used to analyze whether ACEs total score was associated with IWS, as measured by WBIS—M scores, after adjusting for covariates. The overall Step 2 model (including ACEs and covariates) was significant (*F*(4, 44) = 4.40, *p* = 0.005, *R*^2^ = 0.305). Having a higher number of ACEs was significantly associated with higher WBIS—M scores (*β* = 0.40, *p* = 0.006), and ACE score explained 14.6% of the variance in WBIS—M scores. Of the three covariates, only age was found to be significantly associated with WBIS—M scores (*β* = 0.37, *p* = 0.008), suggesting that younger individuals report a greater degree of IWS (see Table 2).

#### 3.2.2. ACE Type Subscale Scores

A second linear regression was conducted to explore differential relationships between ACE-type subscales and IWS, as measured by WBIS—M scores. Multicollinearity checks confirmed the appropriateness of simultaneously including ACE-type subscales in a single model. Results indicated that the overall Step 2 model (including ACE types and covariates) was significant (*F*(6, 44) = 4.66, *p* = 0.001, *R*^2^ = 0.424). Altogether, the three ACE-type subscales explained 26.5% of the variance in WBIS—M scores. However, the Abuse subscale was the only ACE type to be uniquely and significantly associated with WBIS—M scores (*β* = 0.48, *p* = 0.009). Associations between WBIS—M scores and Neglect (*β* = 0.20, *p* = 0.173) or Household Dysfunction (*β* = −0.16, *p* = 0.273) did not reach significance. Younger age was associated with higher WBIS—M scores (*β* = −0.41, *p* = 0.003); neither race nor BMI displayed a significant relationship with WBIS—M scores. These results indicate that individuals who experience abuse-type ACEs and younger individuals reported higher levels of IWS (see Table 2).

### 3.3. Aim 2: Experienced Weight Stigma

#### 3.3.1. Total ACE Score

To examine the association between ACEs history and experienced weight stigma, we conducted a logistic regression with whether or not an individual had ever experienced weight stigma (yes/no) as the dependent variable. Specifically, we evaluated if the total number of ACEs was associated with the odds of experiencing weight stigma, adjusting for age, race, and BMI. The overall Block 2 model (including ACEs and covariates) was significant (χ^2^(4) = 16.55, *p* = 0.002), although ACE score was not a significant predictor (Wald = 1.36, *p* = 0.244). That is, a higher ACE score was not associated with greater odds of experiencing weight stigma (OR = 1.324, 95%CI = 0.825–2.125). However, a descriptive pattern did emerge such that reported rates of lifetime experienced weight stigma were higher in those with high-3+ ACEs (81%) versus medium—1–2 ACEs (67%) or low/no-0 ACEs (60%) (see Table 1).

#### 3.3.2. ACE Type Subscale Scores

An analogous logistic regression analysis was conducted to evaluate whether ACE-type subscale scores predicted the likelihood of experiencing weight stigma. Multicollinearity checks confirmed the appropriateness of simultaneously including ACE-type subscales in a single model. Logistic regression results indicated that although the overall Block 2 model (including ACE types and covariates) was significant (χ^2^(6) = 17.23, *p* = 0.008), no ACE types were significantly associated with experienced weight stigma. Specifically, Wald statistics for the three ACE type subscales were as follows: Abuse (Wald = 0.03, *p* = 0.854), Neglect (Wald = 0.85, *p* = 0.356), and Household Dysfunction (Wald = 1.11, *p* = 0.293). Therefore, neither Abuse (OR = 0.913, 95%CI = 0.346–2.405), Neglect (OR = 1.965, 95%CI = 0.469–8.239), nor Household Dysfunction (OR = 1.526, 95%CI = 0.694–3.356) was associated with the odds that an individual had ever experienced weight stigma.

## 4. Discussion

In a pilot sample of women with overweight/obesity, having a higher number of ACEs was associated with greater endorsement of internalized weight stigma (IWS). The relationship between ACEs and IWS was driven predominantly by experiences of abuse. Though associations between childhood adversity and the internalization of weight stigma have not been thoroughly investigated, previous positive bivariate correlations between ACEs and IWS have been observed [18,19]. The current study replicates and extends these findings by observing that ACEs are predictive of IWS after adjustment for age, race, and BMI. This study also adds to the evidence by showing that ACEs subtypes are differentially associated with IWS, with abuse potentially driving the ACEs–IWS relationship. Childhood physical, sexual, or emotional abuse often includes pervasive and unpredictable exposure to an acute stressor, which may result in more toxic stress and increased allostatic load, as well as having particular impacts on cognitive and emotional patterns (e.g., tendencies toward self-deprecation and shame), as compared to the other ACE types [12,37]. A post hoc sensitivity analysis exploring differences between abuse types (i.e., emotional, sexual, physical) revealed differences such that emotional and sexual abuse were significantly associated with greater IWS (*β*s = 0.380, 0.318; *p*s < 0.05), whereas physical abuse was not (*β* = 0.031, *p* = 0.85) Additional research on the unique effects of each individual ACE and their pathways to adverse outcomes for health and well-being throughout life is warranted.

The observed association between ACEs and IWS provides preliminary support for the theorized cycle between ACEs, negative cognitive/emotional responses related to shame/self-deprecation, and IWS. Based on this cycle, self-critical thoughts and feelings of shame may be potential mechanisms between ACEs and IWS that warrant further investigation. Emerging evidence has identified shame and (lack of) self-compassion as factors that may mediate relationships between IWS and negative outcomes (e.g., emotional eating, depression, anxiety), and negative self-evaluation has been proposed as a critical maintaining factor in IWS [13,14,18–20]. Therefore, after confirming these mechanisms with additional research, targeting these negative cognitive and affective patterns that can develop after experiencing ACEs may be a particularly important point of intervention for reducing the impact of early life adversity on IWS. Additionally, minimizing IWS for persons with a history of ACEs is imperative for promoting their long-term health and well-being. As some experts have suggested that IWS itself may be a chronic stressor [38], evidence-based stress-reduction techniques such as mindfulness and mindfulness-based therapies could be of particular relevance.

Of note, this pilot study did not observe significant relationships between ACEs and lifetime weight stigma experiences. This null finding contrasts previous investigations documenting associations between childhood adversity and greater self-reported frequency of experienced weight stigma [18,19,22]. Potential reasons for this discrepancy include limited power and methodological differences compared to previous investigations (e.g., childhood maltreatment vs. ACEs assessment, past-year weight discrimination vs. lifetime weight stigma experiences). Of note, at the descriptive level, greater rates of experienced weight stigma were endorsed by those with high-3+ ACEs (81%) vs. medium-1–2 ACEs (67%), or low/no-0 ACEs (60%), which suggests that additional investigations in larger samples are warranted.

Additional limitations that warrant follow-up include a relatively homogenous sample (i.e., adult, primarily white, treatment-seeking women with overweight/obesity), which limits generalizability to the larger population (e.g., adolescents and young adults, marginalized individuals). Further, with a small pilot sample, the current study is vulnerable to restriction of range in addition to lack of power. Over two-thirds (71%) of the sample endorsed ever experiencing weight stigma, and this limited variability may have further exacerbated difficulties achieving sufficient power to detect a significant effect of ACEs history on weight-stigma experiences. However, these preliminary results justify future, larger-scale studies with the resources to recruit larger, more diverse samples to more thoroughly investigate relationships between early life stressors, such as ACEs, and weight stigma.

In sum, the current study was a pilot investigation of ACEs and weight stigma in adult women with overweight/obesity. Experiencing a greater number of ACEs in childhood was associated with internalizing weight stigma to a greater degree in adulthood. This finding highlights childhood adversity as an early life factor that may contribute to internalized weight stigma in adulthood. Future research should confirm these relationships and identify mechanisms between experiences of adversity in childhood and adult experiences and the internalization of weight stigma.

## Supplementary Material

Supplementary Material

## Figures and Tables

**Table 1.** Participant characteristics for the full sample and by ACE status.

ACE Status ^a^					
	Total Sample (*N* = 53)	Low/No 0 ACE (*n* = 11)	Medium 1–2 ACE (*n* = 17)	High 3+ ACE (*n* = 23)	*p* ^b^
*M* ± SD or *n* (%)					
*Demographic Factors & Covariates*					
Age (years)	33.19 ± 10.0	33.18 ± 11.2	29.94 ± 8.3	33.74 ± 10.1	0.314
BMI (kg/m^2^)	33.70 ± 7.2	32.35 ± 7.3	34.93 ± 7.9	32.89 ± 5.9	0.563
Race/Ethnicity					
Non-Hispanic white	36 (67.9)	10 (90.9)	12 (70.6)	14 (60.9)	0.198
Historically marginalized/oppressed racial group	17 (32.1)	1 (9.1)	5 (29.4)	9 (39.1)	
*Key Study Variables*					
WBIS—M (1–7)	4.59 ± 0.9	4.11 ± 1.0	4.49 ± 0.9	4.83 ± 0.9	0.108 ^†^
Experienced Weight Stigma (Y/N)	34 (70.8)	6 (60.0)	10 (66.7)	17 (81.0)	0.417
ACE Questionnaire Total Score (0–17)	2.90 ± 2.9	–	1.50 ± 0.7	5.26 ± 2.5	-
ACE Subtype Frequency					
Abuse	28 (52.8)	–	9 (52.9)	19 (82.6)	0.072 ^†^
Neglect	18 (34.0)	–	2 (11.8)	15 (65.2)	0.001 *
Household Dysfunction	32 (60.4)	–	9 (52.9)	22 (95.7)	0.001 *

Note. Continuous variables are presented as *M*(SD). Categorical variables presented as *n*(%).

a Based on collapsed 10-item ACE scores.

b *p* represents differences between ACE status based on a one-way ANOVA for continuous variables and a Chi-square for categorical variables. Only those with Medium and High ACEs were included in Chi-squares for ACE subtypes.

* Significant at *p* < 0.05.

† trending toward significance at *p* < 0.15. ACE = Adverse Childhood Experiences. WBIS—M = Weight Bias Internalized Scale—Modified.

**Table 2.** Associations of adverse childhood experiences with internalized weight stigma.

	Model 1a Total ACEs Predicting WBIS—M Scores (*n* = 46)		Model 1b ACE-Type Subscales Predicting WBIS—M Scores (*n* = 46)	

	***R^2^***	**Δ*R^2^***	***R^2^***	**Δ*R^2^***
Step 1 ^†^	0.159	–	0.159	–
Step 2	0.305	0.146 *	0.424	0.265 *

	***β***	***p***	***β***	***p***
Age	0.373 *	0.008	0.407 *	0.003
Race/Ethnicity ^‡^	0.181	0.190	0.252	0.059
BMI	0.218	0.106	00.166	0.191
**Total ACE** **Questionnaire Score**	0.399 *	0.006	-	-
**ACEs—Abuse**	-	-	0.477 *	0.009
**ACEs—Neglect**	-	-	0.203	0.173
**ACEs—Household** **Dysfunction**	-	-	−0.161	0.273

Note:

* *p* < 0.05

† Only covariates (age, race/ethnicity, BMI) were entered on Step 1. For parsimony, specific beta coefficients of covariates are only presented for Step 2

‡ 0 = oppressed/marginalized racial group, 1 = non-Hispanic white; ACE = adverse childhood experience; ACEs—Abuse = emotional, physical, or sexual abuse subscale score; ACEs—Neglect = emotional or physical neglect subscale score; ACEs—Household Dysfunction = mother treated violently, substance abuse, or mental illness in the household, parental separation or divorce, or incarcerated household member subscale score; BMI = body mass index; WBIS—M = Weight Bias Internalization Scale—modified.

## Data Availability

Data are not publicly available but interested parties may contact the corresponding author for inquiries.
