# Supplementary material for: Adverse Childhood Experiences Associated with Greater Internalization of Weight Stigma in Women with Excess Weight
Source: Obesities. Author manuscript; Available in PMC 2022 Jun 3. (PMC9033161; doi:10.3390/obesities1010005)
Supplement: Supplementary Material [file NIHMS1742379-supplement-Supplementary_Material.pdf]

Table S1

Adverse Childhood Experiences Questionnaire Subscale Items

ACEs-Abuse

1) Did a parent or other adult in the household often swear at you, insult you, put you down, or humiliate you?
2) Did a parent or other adult in the household often act in a way that made you afraid that you might be physically hurt?
3) Did a parent or other adult in the household often push, grab, slap, or throw something at you?
4) Did a parent or other adult in the household ever hit you so hard that you had marks or were injured?
5) Did an adult or person at least 5 years older than you ever touch or fondle you or have you touch their body in a sexual way?
6) Did an adult or person at least 5 years older than you ever try to or actually have oral, anal, or vaginal sex with you?

ACEs-Neglect

7) Did you often feel that no one in your family loved you or thought you were important or special?
8) Did you often feel that your family didn't look out for each other, feel close to each other, or support each other?
9) Did you often feel that you didn't have enough to eat, had to wear dirty clothes, and had no one to protect you?
10) Did you often feel that your parents were too drunk or high to take care of you or take you to the doctor if you needed it?

ACEs-Household Dysfunction

11) Were your parents ever separated or divorced?
12) Was your mother or stepmother often pushed, grabbed, slapped, or had something thrown at her?
13) Was your mother or stepmother sometimes or often kicked, bitten, hit with a fist, or hit with something hard?
14) Was your mother or stepmother ever repeatedly hit over at least a few minutes or threatened with a gun or knife?
15) Did you live with anyone who was a problem drinker or alcoholic or who used street drugs?
16) Was a household member depressed or mentally ill or did a household member attempt suicide?
17) Did a household member ever go to prison?

Note: ACE=adverse childhood experience
